# Association Between Self-Reported Adherence to Preventive Practices and Probability of Turning COVID-19 Positive: A Cross-Sectional Analytical Study

**DOI:** 10.7759/cureus.11815

**Published:** 2020-12-01

**Authors:** Piyush Ranjan, Aakashneel Bhattacharya, Avinash Chakrawarty, Rojaleen Das, Arvind Kumar, Shivam Pandey, Souradeep Chowdhury, Ankit Mittal, Upendra Baitha, Naveet Wig

**Affiliations:** 1 Medicine, All India Institute of Medical Sciences, New Delhi, IND; 2 Infectious Diseases, All India Institute of Medical Sciences, New Delhi, IND; 3 Geriatric Medicine, All India Institute of Medical Sciences, New Delhi, IND; 4 Microbiology, All India Institute of Medical Sciences, New Delhi, IND; 5 Biostatistics, All India Institute of Medical Sciences, New Delhi, IND; 6 Internal Medicine, All India Institute of Medical Sciences, New Delhi, IND

**Keywords:** covid-19, preventive practices, odds ratio, pandemic, risk factors

## Abstract

Background

Preventive practices are the mainstay to mitigate the spread of the COVID-19 pandemic. We tried to assess the self-reported adherence of our participants to the already known preventive practices. Furthermore, we tried to determine whether the non-compliance to specific preventive practices was associated with the acquisition of the infection or not.

Methods

We enrolled 379 healthcare workers, hospital staff, and their family members who were tested for COVID-19 by reverse transcription-polymerase chain reaction (RT-PCR) in an outpatient clinic. Socio-demography and the infection prevention practices of the individuals were recorded in a preformed questionnaire. Statistical analysis was performed to find out the statistical association between these factors and the RT-PCR results. Adjusted and unadjusted odds ratios were determined to find out the degree of protection provided by each of the preventive practices concerning the development of the disease.

Results

Social distancing (p<0.001), hand hygiene (p<0.001), ensuring N-95 mask fit check (p<0.001), and the use of alternative medications (p=0.002) were found to be protective. Resident doctors were at a lower risk of developing the disease as compared to the other healthcare workers (odds ratio: 0.39).

Conclusion

The failure to practice the already known preventive practices is probably one of the most important factors in the progression of the COVID-19 pandemic. Adherence to these practices is the intervention of choice to reduce disease transmission in the current scenario.

## Introduction

This century has witnessed the biggest global health problem that has affected every aspect of human life. Coronavirus disease (COVID-19) pandemic has already claimed almost 1.2 million lives all over the world, and 47 million people have been infected by the severe acute respiratory syndrome coronavirus 2 (SARS-CoV-2) till November 2, 2020 [1]. To date, no reliable treatment or vaccine is available for the control of the disease, and thus adherence to preventive measures are the most important interventions to control the disease.

Several guidelines have recommended using masks, a physical distancing of more than six feet, and hand hygiene as prevention practices against COVID-19, which are effective in breaking the chain of transmission [2, 3]. Although most people are aware of these infection prevention practices, it is not certain what proportion of people comply with these practices in their day to day lives. Whether a lapse in these preventive practices is actually associated with an increased chance of being infected by SARS-CoV-2 needs to be investigated.

This study was conducted in an outpatient setting at a tertiary care hospital in New Delhi, India, to study the self-reported compliance to the preventive practices followed by the general public as well as the healthcare workers who visited the clinic to get themselves tested for COVID-19. The study's objective was to determine whether a specific socio-demographic factor or preventive practice was associated with the probability of a person being COVID-19 positive.

## Materials and methods

We conducted a single-center cross-sectional study among the attendees of an outpatient COVID-19 clinic at a tertiary care hospital in New Delhi, India. This clinic is run for providing healthcare services to the workers who are under institutional health schemes. The institute ethics committee approved the study protocol, and appropriate consent was taken from the participants before their enrolment.

Between June 17 to July 1, 2020, 1,066 patients were tested for SARS-CoV-2 by reverse transcription-polymerase chain reaction (RT-PCR) in the screening clinic. Three hundred and eighty-four patients were included in the study based on successful telephonic communication and informed consent availability. Five patients were excluded from the study due to the non-availability of their test results due to pre-analytical issues. We analyzed socio-demographic and infection prevention practices related to data collected from 379 patients. Each patient was telephonically interviewed in the local language by a single investigator, and the answers were documented in a preformed questionnaire proforma. To minimize bias, the telephonic communications were completed before their test results were generated.

Study participants were tested for COVID-19 after a thorough clinical examination. Real-time RT-PCR for SARS-CoV-2 was performed for the diagnosis of COVID-19 in all patients included in our study. Testing indications were based on the national regulatory authority's advisory, the Indian Council of Medical Research (ICMR) [4]. One nasal and one throat swab were collected from each patient. Two swabs were put into a single vial of the viral transport medium (VTM) and were sent to the testing laboratory maintaining the cold chain. The test reports were accessed from the hospital information system.

A questionnaire was developed to collect socio-economic, demographic, behavioral, and infection prevention practices information from the enrolled participants (Table 1). Socio-economic status was documented as per the modified Kuppuswamy index [5]. More than three individuals residing in one room were considered as overcrowding [6]. In India, ICMR, the national regulatory authority, has recommended prophylaxis with hydroxychloroquine for workers who are at occupational risk of acquiring this infection [7]. Therefore, the healthcare workers were enquired about the use of hydroxychloroquine for prophylaxis against COVID-19. The prophylactic use of other medications (alternative medicines) was also recorded from the study population.

**Table 1:** Risk factors and preventive practices enquired from the participants PPE - personal protective equipment

Parameters evaluated	Questions asked
Commute to workplace	Do you commute to your workplace alone or do you use public transport?
	Yes
	No
Hydroxychloroquine prophylaxis	Are you taking hydroxychloroquine tablets for prophylaxis against COVID-19?
	Yes
	No
Social distancing	How frequently do you maintain social distancing which means staying 6 feet away from each other?
	Almost always (more than 90%)
	Mostly (about 75%)
	Commonly (about 50%)
	Sometimes (about 25%)
	Rarely (less than 10%)
Hand hygiene	How frequently do you wash your hands with soap water or alcohol-based hand rubs?
	Once every hour
	Every 2-3 hourly
	Every 3-4 hourly
	Less than that
	Do you ensure that you spend 40 seconds every time washing your hands?
	Yes
	No
Use of PPE by healthcare workers	What PPE do you use while working in the healthcare setting?
	Mask
	Gown
	Gloves
	Cap
	Shoe covers
	Goggles/ Eyeshield

Statistical analysis

Data was recorded on a pre-designed proforma and managed in an excel spreadsheet. All the entries were checked for any possible keyboard errors. Quantitative variables were assessed for approximate normality and summarized as mean ± SD or median (Q1, Q3). In step 1, logistic regression analysis was used with the outcome and each of the independent variables separately. In step 2, multiple logistic regression analysis was performed with all the variables simultaneously with the outcome variable separately. Stata 15.0 statistical software (StataCorp LLC, College Station, USA) was used for data analysis. In this study, a p-value <0.05 was considered as statistically significant.

## Results

Among 379 participants, 245 (64.6%) were males, and 134 (35.3%) were females. The mean age of the participants was years 35.2 ± 11.3 (SD). Most of them (37.9%) belonged to upper-lower socioeconomic status, followed by lower (20.8%), upper (14.5%), lower-middle (14.2%), and upper-middle (11.3%) categories as per the Kuppuswamy socioeconomic scale (Table 2). Statistical analysis of these findings did not reveal any association between age, gender, and socioeconomic status with the COVID-19 positive test result.

**Table 2:** General characteristics and risk factors of the study population RT-PCR - reverse transcription-polymerase chain reaction

General characteristics	RT-PCR positive n=126 (33.2%)	RT-PCR negative n=253 (66.8%)	p-value
Mean age (in years) ±SD	36.6 ± 12.4	34.5 ± 10.7	0.09
Sex			0.7
Male	80 (63.5)	165 (65.2)	
Female	46 (36.5)	88 (34.8)	
Socioeconomic status (Kuppuswamy index)			0.610
Upper	18 (14.5)	37 (14.7)	
Upper middle	17 (13.7)	26 (10.4)	
Lower middle	21 (16.9)	33 (13.1)	
Upper lower	46 (37.1)	98 (39.0)	
Lower	22 (17.7)	57 (22.7)	
Occupation			0.009
Doctor/ Faculty	3 (2.4)	4 (1.6)	
Doctor/ Resident	16 (12.7)	78 (31.2)	
Nursing officer	8 (6.3)	10 (4.0)	
Laboratory technician	6 (4.8)	10 (4.0)	
OT technician	6 (4.8)	9 (3.6)	
Security guard	6 (4.8)	3 (1.2)	
Housekeeping staff	20 (15.9)	31 (12.4)	
Others	61 (48.4)	108 (43.2)	
Overcrowding at home	17 (13.5)	39 (15.4)	0.619
Mode of commute to workplace			0.452
Alone	19 (15.1)	44 (17.4)	
Public transport with single occupancy	2 (1.6)	9 (3.6)	
Public transport with multiple occupancy	105 (83.3)	200 (79.1)	

Out of 379 patients, 126 (33.2%) were detected to be COVID-19 positive. Two hundred and one (53%) participants were healthcare workers, and 94 (46.7%) of them were the resident doctors who had been tested for COVID-19 (Table 2). Occupation of the patients was statistically associated with the development of the disease (p=0.009). It was found that the resident doctors were at the least risk to develop the disease (odds ratio: 0.39) (Table 3).

**Table 3:** Adjusted and unadjusted odds ratios against the risk factors

Risk factors	Unadjusted odds ratio (95% CI)	Adjusted odds ratio (95% CI)
Social distancing of six feet or more		
Almost always (more than 90%)	1	1
Commonly (about 50%)	0.32 (0.10-0.97)	0.15 (0.07-0.32)
Sometimes (about 25%)	0.40 (0.01- 0.21)	0.08 (0.03-0.19)
Rarely (less than 10%)	0.20 (0.03-1.21)	0.15 (0.05-0.46)
Hand hygiene frequency		
Once every hour	1	1
Every 2-3 hourly	0.80 (0.24-2.68)	0.36 (0.15-0.82)
Every 3-4 hourly	0.35 (0.11-1.10)	0.08 (0.03-0.22)
Less than that	0.25 (0.07-0.80)	0.14 (0.06-0.34)
Ensuring N-95 mask fitness	2.04 (0.83-4.99)	0.35 (0.18-0.66)
Resident doctors	0.27 (0.05-1.34)	0.39 (0.19-0.79)

Participants were enquired about their living conditions at their residence, and only 56 (14.7%) of them gave a history of overcrowding at home. A large proportion of the participants (n=305, 80.4%) used public transport with multiple occupancies to commute to their workplace in this pandemic situation. Among the rest, 16.6% used to commute alone either by walking or in their own vehicles, and only 3% traveled to their workplace in public transports with single commuter options (Table 2). Overcrowding at home and the nature of commuting to the workplace did not reveal any statistical correlation with the acquisition of the disease.

We enquired about the medications that they were taking for prophylaxis against COVID-19. Eighty-one patients (21.3%) took hydroxychloroquine prophylaxis as per India's national guidelines [7]. Some of the participants (30.3%) used alternative medicines as prophylaxis against COVID-19 (Table 4). These traditional medicines are used for ages in this part of the world for their possible role in promoting immunity against infections [8]. The use of hydroxychloroquine was not found to be protective (p=0.059), while the alternative medicines have shown to be protective against COVID-19 (p=0.002) while used prophylactically. Only sixty-seven of the participants (17.7%) followed social distancing of six feet or more at more than 90% of occasions, and 61.5% of the patients followed the same at 50% or less of the occasions (Table 4). When inquired about hand hygiene practices, 144 (37.9%) of respondents performed hand washing (either with alcohol-based hand rub or soap water) once in every 2-3 hours intervals. The appropriate duration of handwashing was ensured by only 118 (31.1%) patients. Almost all of the participants used N-95 masks routinely, but only 86 (22.6%) used their masks after an appropriate fit check (Table 4). We observed that maintaining social distancing of six feet or more, practicing hand hygiene, ensuring proper duration of handwashing, and adhering to N-95 mask fit checks were statistically significant (p<0.001) with the prevention of transmission of SARS-CoV-2 infection.

**Table 4:** Reported compliance with preventive practices against COVID-19 RT-PCR - reverse transcription-polymerase chain reaction

Preventive practices	RT-PCR positive n=126 (33.2%)	RT-PCR negative n=253 (66.8%)	p-value
Use of drugs for COVID-19 prophylaxis			
Hydroxychloroquine	20 (16.3)	61 (24.9)	0.059
Alternative medicines	52 (41.9)	63 (26.2)	0.002
Practice social distancing of staying 6 feet away from each other			< 0.001
Almost always (more than 90%)	42 (33.3)	25 (9.9)	
Mostly (about 75%)	33 (25.2)	46 (18.1)	
Commonly (about 50%)	25 (19.8)	68 (26.9)	
Sometimes (about 25%)	17 (13.5)	90 (35.6)	
Rarely (less than 10%)	9 (7.1)	24 (9.4)	
Practiced mask fit check	42 (33.3)	44 (17.4)	< 0.001
Hand hygiene			
Frequency			< 0.001
Once every hour	38 (32.8)	26 (10.3)	
Every 2-3 hourly	30 (25.9)	50 (19.7)	
Every 3-4 hourly	21 (18.1)	77 (30.4)	
Less than that	37 (31.9)	100 (39.5)	
Appropriate duration ensured	59 (46.8)	59 (23.3)	< 0.001

In order to understand the protective efficacy of personal protective equipment (PPE), we also assessed the appropriate usage of PPE and different PPE kits from the answers gathered from 150 of the participants (doctors, nursing staff, laboratory technicians, and the OT technicians) (Table 5). The use of face shields for eye protection as a part of other PPE components was significantly associated (p=0.021) with a reduced chance of risk of acquiring the infection.

**Table 5:** PPE use among health care workers PPE - personal protective equipment; RT-PCR - reverse transcription-polymerase chain reaction

Use of PPE during working in hospital by healthcare workers (n = 150)	RT-PCR positive n=39 (26%)	RT-PCR negative n=111 (74%)	p-value
N-95 mask with fit check	18 (46.2)	67 (60.4)	0.124
Gown	16 (41.0)	57 (51.4)	0.267
Goggles	8 (20.5)	41 (36.9)	0.060
Face shield	10 (25.6)	52 (46.8)	0.021
Cap	11 (28.2)	49 (44.1)	0.080
Shoe covers	12 (30.8)	48 (42.3)	0.171

The unadjusted and adjusted odds ratios were calculated for preventive practices, which had a statistical influence on the development of COVID-19 (Table 5). The practice of social distancing of more than six feet, maintenance of hand hygiene, ensuring the appropriate duration of hand washings, compliance with N-95 mask fit checks were found to be protective when analyzed against COVID-19 test results.

## Discussion

COVID-19 has already taken a great toll on human lives and has disrupted human routines in an unprecedented manner [9]. Healthcare systems are devastated by the increasing number of cases, and the healthcare workers are exposed to the highest risk of contracting the infection. Infection prevention practices are the mainstay of minimizing the risk of transmission, helping in mitigating the spread of this pandemic. In this study, we have analyzed the preventive practices of patients presenting to an outpatient clinic for the testing for SARS-CoV-2. The self-reported compliance was statistically analyzed with the COVID-19 RT-PCR reports to observe the association between the adherence to the preventive practices and their COVID-19 test results.

There are some significant findings in this study. In the analysis of the socio-demographic features, there was no statistical correlation between the individuals' age, gender, socioeconomic status, and chances of acquiring COVID-19. Though there are conflicting reports of age and gender as risk factors for acquiring the disease, the pandemic has crossed geographical and socio-economic boundaries to infect citizens at an exponential rate [10-12]. The resident doctors were at a lower risk of contracting the infection when compared with other healthcare workers. They are the frontline workers in our hospital, being at the highest risk of exposure due to the nature of the work they use to perform, including aerosol-generating procedures, which carry the highest risk for transmission of the disease [3]. We assume that they adhered to the infection prevention practices to the maximum extent, leading to a lower incidence of COVID-19 amongst them while being present at the forefront.

The prophylactic use of hydroxychloroquine was associated with some protection level (p=0.059), which was not statistically significant. With its in-vitro effectivity against SARS-CoV-2 and good lung concentration, hydroxychloroquine is a drug candidate selected to be used as prophylaxis against COVID-19 [13-15]. But the actual protection level is still unknown, and it is to be established by larger studies. It was found in the study that the alternative medicines were effective against COVID-19 while taken as prophylaxis. These medications are considered as immunity boosters and used by a larger portion of society in this subcontinent, though the actual form of the alternative medications was not enquired of in this study. We do not conclude about the prophylactic role of these medications here as the possible reason for the apparent protection conferred by the alternative medications might lie in the fact that people exercising such measures are more conscious about other preventive practices like mask use and social distancing, which are proven to be effective in preventing the transmission of COVID-19.

The social distancing of more than six feet was found to be statistically relevant as a protective measure against COVID-19 and should be practiced at all times. There is an established role of social distancing to mitigate the spread of the disease. A systematic review had concluded that the transmission of the virus becomes significantly lower when a physical distancing of 1 meter (~ three feet) or more was maintained (n= 10,736, pooled adjusted odds ratio 0.18, 95% CI 0.09-0.38) [2]. The authors have also shown that the degree of protection improves if the distance is increased. 

It was not surprising that participants who reported to wash their hands at regular intervals while maintaining appropriate duration and who used their N-95 masks with recommended fit check were protected against this disease. The practice of hand hygiene has been strongly recommended by the World Health Organisation (WHO) both for the general public and the health care workers as a part of contact precaution against COVID-19 [3].

While analyzing the practice of using PPE and PPE kits among frontline healthcare workers (n=150), we did not find any significant statistical correlation between their use and the chances of acquiring COVID-19 except for the use of face shields, which were found to be protective. But no concluding remarks could not be made owing to the smaller sample size.

The study's major strength is that all the samples were tested by RT-PCR, which is considered the gold standard for the diagnosis of COVID-19. This study is one of the first studies from India to report a direct association with preventive practices with the COVID-19 test results.

This study design was based on telephonic interviews, which might be responsible for some degree of recall bias. There is some missing data for some of the participants, mainly due to network outages during the telephonic communications. Furthermore, it was performed in a single center involving a specific population of patients; thus, the results may not be considered a reflection of the general population's behavioral practices.

There is a considerable amount of fear associated with this disease due to its rapid spread, high transmissibility, and substantial mortality in the susceptible population. The general public is already aware of the infection prevention practices against COVID-19, which are already circulated in the news, printed, and social media. There are several numbers of studies all over the world regarding the knowledge, attitude, and practices of the general population and healthcare workers during this pandemic [16-18]. But, it has been observed in other studies that people actually fail to practice the preventive measures in a consistent manner, which is largely responsible for the progression of this pandemic [19]. Thus, religious adherence to the preventive practices by the general public and healthcare workers can help implement proper preventive strategies in the current global scenario.

## Conclusions

It is beyond any doubt that we have to adhere to the infection prevention practices against COVID-19 till an effective and safe vaccine becomes available to the general public. Our study shows that it is not the socio-demographic factors but the lack of preventive practices that are statistically associated with the development of the disease among the attendees of a COVID-19 clinic in an urban setting. The social distancing of more than six feet, maintaining hand hygiene at frequent intervals, and ensuring N-95 mask fitness were three major practices that showed protection against the disease when analyzed statistically. Pre-exposure prophylaxis with hydroxychloroquine did not show any protective role, and the prophylactic role of the alternative medicines against COVID-19 is unclear in this study. Finally, the preventive practices go hand in hand with the awareness against the disease, and they are only effective when followed in every aspect of our lives. Adherence to these practices is still not satisfactory; thus, behavioral modifications are required in the general public's day-to-day lives to follow preventive practices consistently.
